# The Sediment–Water Partitioning Characteristics of Per- and Polyfluoroalkyl Substances (PFAS) in Urban Rivers Receiving Reclaimed Water

**DOI:** 10.3390/toxics14030190

**Published:** 2026-02-25

**Authors:** Yuhan Gao, Zhaohe Zhang, Dian Chen, Yue Lan, Li Wang, Xingchun Jiao

**Affiliations:** 1 National Research Center for Geoanalysis, Chinese Academy of Geological Sciences, Beijing 100037, China; gaoyuhan23@mails.ucas.ac.cn (Y.G.); 17879506126@163.com (Y.L.); 2 Land Space Ecological Restoration Center of Jining City, Jining 272000, China; 15623635977@163.com; 3 Yunnan Geological Engineering Second Survey Institute Company Limited, Kunming 650218, China; chendianbj@163.com; 4 China Institute of Water Resources and Hydropower Research, Beijing 100038, China; wangli@iwhr.com

**Keywords:** urban runoff, per- and polyfluoroalkyl substances (PFAS), sediment–water partition, reclaimed water

## Abstract

Urban rivers often contain a complex mixture of contaminants including per- and polyfluoroalkyl substances (PFAS), metals, and various salts. This study aimed to investigate the sediment–water partitioning characteristics of PFAS in urban rivers and analyze the hydrochemical causes of this specific feature. We sampled paired water and sediment samples from urban rivers in a reclaimed water irrigation area in Beijing City. The average total PFAS concentrations in the river water and sediment were 28.44 ± 16.37 ng/L and 6.41 ± 4.20 ng/g dw, respectively. Short-chain PFAS from C4 to C6 and PFCA congeners dominated in the water, while long-chain PFAS above C8 and PFSA congeners dominated in the sediment. The average sediment–water ratio (Log *K_d_*) of PFAS at each site showed an increasing trend with chain length, and was generally higher than that observed in seawater, natural rivers, and lakes, indicating a specific sediment–water partitioning behavior of PFAS in urban rivers. This difference is likely due to the distinct hydrochemical characteristics of the urban rivers, where elevated TDS, the presence of surfactants, and the coexistence of multiple heavy metal ions collectively promote PFAS adsorption onto suspended particulate matter and enhance their accumulation in sediments through sedimentation.

## 1. Introduction

Clean drinking water and domestic water supplies are essential for human health and sustainable development. With the continued expansion of urbanization, water scarcity has increased, accompanied by growing concern over the quality of urban water environments [1]. Urban runoff and reclaimed water play an important role in water storage, irrigation, and the regulation of groundwater to cities [2]. However, urban runoff collects municipal sewage and industrial wastewater along its flow path, resulting in severe water quality degradation. This is manifested by ammonia nitrogen pollution, elevated toxic heavy metal concentrations, and frequent detection of various organic pollutants [3,4]. Among these contaminants, per- and polyfluoroalkyl substances (PFAS), as a class of contaminants of emerging concern, have attracted considerable attention in water environments because of their extreme environmental persistence and potential health risks [5,6]. Due to the C-F bond being a highly covalent bond, conventional water treatment processes are generally ineffective at cleaving C–F bonds [7,8]. As a result, PFAS precursors may be transformed during treatment, leading to an increase rather than a reduction in PFAS concentrations in the treated effluent [9]. Zhang [10] compared the removal efficiencies of per- and polyfluoroalkyl substances (PFAS) in wastewater treatment plants (WWTPs) located in Tianjin, Shenyang, and Fuxin. The study found that neither the sedimentation tank nor the continuous microfiltration processes achieved significant removal of PFAS. Furthermore, chlorination treatment resulted in increased effluent concentrations for most PFAS compounds. Pan et al. [11] reported that influent PFAS concentrations in seven wastewater treatment plants in Beijing ranged from 2.88 to 176 ng/L, while effluent concentrations ranged from 5.48 to 498 ng/L. On average, the PFAS mass flux in the effluent was approximately 127% higher than that in the influent. While advanced WWTP technologies (e.g., ion-exchange adsorption, electrochemical degradation, nanofiltration) can achieve PFAS removal efficiencies of 95–100% [12], their large-scale application remains limited by practical considerations including cost, operational demands, and the persistent background levels of PFAS in the environment. Such effluent can become a major source of PFAS contamination once discharged into urban rivers [13].

While many existing studies on PFAS in the surface water environment have focused on rivers [14,15], lakes [16,17], and other natural water bodies [18,19], studies addressing the typical characteristics of the sediment–water partitioning of PFAS in urban rivers receiving reclaimed water remain relatively limited. Furthermore, PFAS contamination in urban rivers is becoming increasingly prevalent and typically occurs at ng/L levels. For example, the mean total PFAS concentration in Shanghai’s Huangpu River was reported to be 171.65 ng/L, with values ranging from 113.38 to 362.37 ng/L [20]. In the Qinhuai River of Nanjing, the PFAA concentrations ranged from 0.8 to 95.6 ng/L during the rainy season and from 13.8 to 274.6 ng/L during the dry season [21]. A notably high PFAS concentration of 2234.3 ng/L was detected in a river flowing through the city of Las Vegas [22]. Therefore, it is necessary to investigate the occurrence and distribution features of PFAS in urban rivers to mitigate urban water pollution and safeguard public health.

This study focuses on the occurrence and partitioning characteristics of PFAS in urban rivers located within a reclaimed water irrigation area in suburban Beijing, China. Previous studies have reported the widespread detection of PFAS in both shallow and deep groundwater in this area [5,23]. However, the extent of PFAS pollution in the surface water and its potential implications for groundwater contamination remain poorly documented. We investigated the accumulation and spatial distribution of PFAS in the two surface rivers receiving reclaimed water, specifically analyzed the sediment–water partitioning characteristics of PFAS, and elucidated the differences compared with other water bodies. The results would contribute to a better understanding of the accumulation characteristics of PFAS in the urban surface water environment, prompting the implementation of preventive measures to protect groundwater.

## 2. Materials and Methods

### 2.1. Study Area and Sample Collection

**Figure 1:**
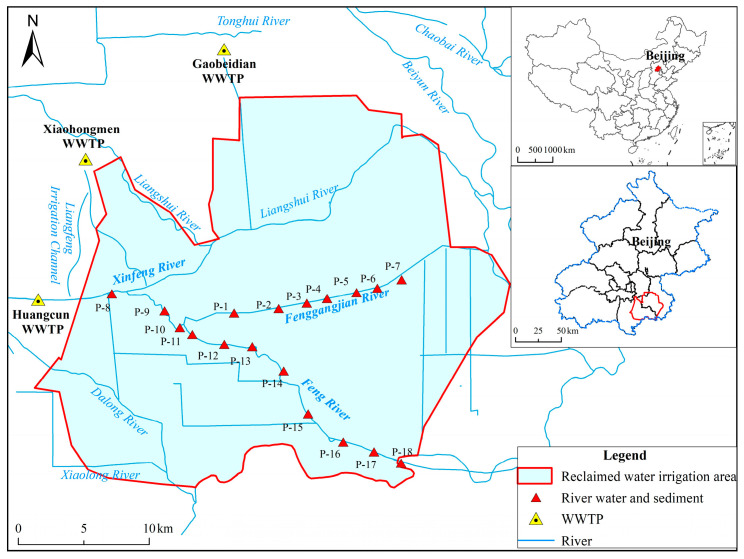
Map of the study area and sampling sites.

The study area is located in the southeastern suburbs of Beijing, China (39°26′ N–39°50′ N, 116°13′ E–116°43′ E), within an irrigation area with a long history of using reclaimed water (Figure 1). Wastewater is conveyed to the irrigation area through river channels and main delivery canals. The reclaimed water is primarily supplied by secondary effluents from three wastewater treatment plants (WWTPs)—Gaobeidian, Xiaohongmen, and Huangcun—with diversion capacities of 105, 150, and 20 million m^3^/yr, respectively. Owing to the continuity and stability of the reclaimed water supply, the irrigation area incorporates wetlands, lakes, and other reclaimed water storage infrastructures to facilitate regulation and utilization [24].

In April 2021, a total of 18 paired river water and sediment samples were collected from the rivers within the irrigation area, with an approximate spacing of 2 km between adjacent sites (Figure 1). River water samples were collected at depths of 0.3–0.5 m below the water surface using stainless-steel buckets, and care was taken to avoid disturbance of bottom sediments to prevent contamination. At each site, duplicate water samples were collected in 500 mL polypropylene bottles. All water samples were transported to the laboratory under refrigerated conditions (4 °C) and stored at the same temperature before analysis. Reagent water was used as a process control to prepare field blanks. Sediment samples were collected from the upper 0–5 cm of the riverbank using handheld shovels. Approximately 500 g of sediment was collected at each site, placed in PP containers, and transported to the laboratory on the same day, where the samples were refrigerated before further analysis.

### 2.2. Experimental Materials

The target analytes comprised 13 PFAS, including nine perfluoroalkyl carboxylic acids (PFCAs), namely PFBA, PFPeA, PFHxA, PFHpA, PFOA, PFNA, PFDA, PFUnDA, and PFDoA, as well as four perfluoroalkyl sulfonic acids (PFSAs), including PFBS, PFHxS, PFOS, and PFDS. The isotopically labeled internal standards used were ^13^C_4_PFBA, ^13^C_2_PFHxA, ^13^C_4_PFOA, ^13^C_5_PFNA, ^13^C_2_PFDA, ^13^C_2_PFUnDA, ^18^O_2_PFHxS, and ^13^C_4_PFOS. All mixed standard solutions were purchased from Wellington Laboratories (Guelph, ON, Canada). Ultrapure water with a resistivity of 18.2 MΩ·cm at 25 °C was used throughout the experiments. HPLC-grade methanol was obtained from Merck (Darmstadt, Germany). Ammonia solution, acetic acid (99.9%), and ammonium acetate (97%) were obtained from Fluka (Ronkonkoma, NY, USA).

### 2.3. Sample Extraction

River water and sediment samples were pretreated following the methods described by Chen et al. [5] and Jiao et al. [23], as summarized below. An Oasis WAX solid-phase extraction cartridge (6 cc, 150 mg, 6 μm; Waters Corp., Milford, MA, USA) was used to extract a 200 mL aliquot of each water sample. Before sample loading, the cartridges were activated with 4 mL of 0.1% ammonia-methanol solution, followed by 4 mL of methanol and 4 mL of Milli-Q water. After sample loading, the cartridges were washed with 4 mL of 25 mmol/L acetate buffer (pH = 4) to remove impurities. The target analytes were sequentially eluted with 4 mL of methanol followed by 4 mL of 0.1% ammonia-methanol solution. The eluate was concentrated to 1 mL under a gentle nitrogen stream and filtered through a 0.22 μm organic phase nylon syringe filter, and then transferred to a polypropylene vial for instrumental analysis. Sediment samples were air-dried and homogenized after sieving through a 100-mesh screen. Approximately 1 g of sediment was ultrasonically extracted using three successive 5 mL aliquots of methanol. After centrifugation, the supernatant was collected, evaporated to 4 mL under high-purity nitrogen, diluted to 200 mL with Milli-Q water, and subjected to solid-phase extraction using an Oasis WAX cartridge. The subsequent extraction procedure was identical to that used for water samples.

### 2.4. Instrumentation and Analysis Conditions

PFAS were analyzed using an Agilent 1200 high-performance liquid chromatograph (Agilent Technologies Inc., Santa Clara, CA, USA) coupled with an API 4000 triple quadrupole tandem mass spectrometer (AB Sciex, Concord, ON, Canada). Chromatographic separation was achieved using an XBridge-C_18_ LC column (150 mm × 4.6 mm, 3.5 μm; Waters, Milford, MA, USA) under negative electrospray ionization mode. The mobile phase consisted of methanol and 2 mmol ammonium acetate and was delivered under gradient elution at a flow rate of 0.3 mL/min. Detection was performed using multiple reaction monitoring (MRM). The detailed mass spectrometric and chromatographic parameters have been reported by Chen et al. [5].

### 2.5. Quality Assurance and Quality Control

To minimize potential PFAS contamination, polytetrafluoroethylene (PTFE)-free laboratory supplies were used throughout sample pretreatment and collection. Target compounds were quantified using an internal standard method. A seven-point calibration curve was established using standard solutions at concentrations of 10, 20, 50, 100, 200, 500, and 1000 ng/L, with linear correlation coefficients (r) ranging from 0.9916 to 0.9996. To evaluate the method accuracy and reliability, matrix recovery tests were conducted at three concentration levels of 100, 500, and 1000 ng/L. To ensure instrumental stability and minimize carryover contamination, one solvent blank and one calibration standard were analyzed after every ten samples. Samples with target compound recoveries outside the acceptable ranges of 60–120% for water and 50–110% for sediment, or with surrogate recoveries outside 60–100%, were reanalyzed. Reported concentrations were not recovery-corrected. Method detection limits and recoveries are summarized in Table 1.

## 3. Results and Discussion

### 3.1. PFAS in River Water and Sediment

**Figure 2:**
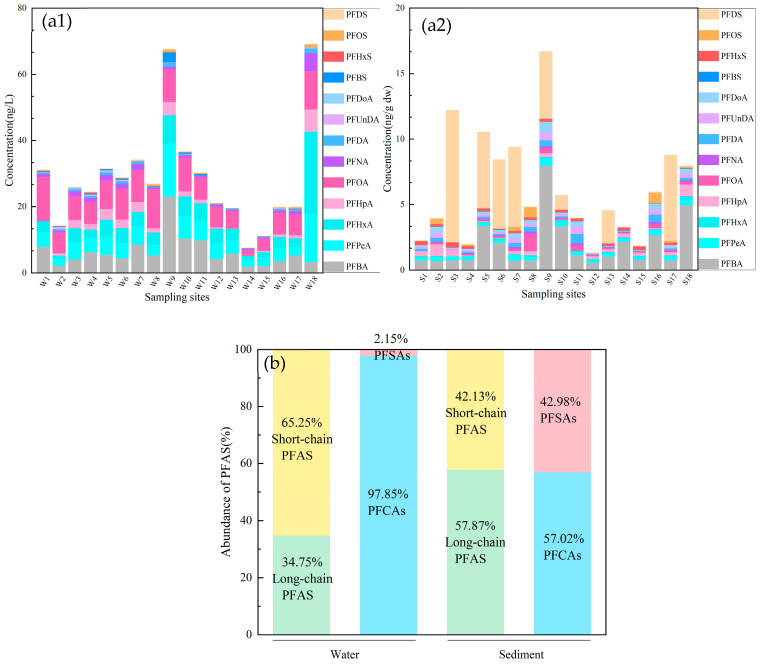
Detection (**a1**,**a2**) and proportion (**b**) of PFAS in river water and sediment.

A total of 13 target PFAS were detected in river water samples, with total concentrations ranging from 7.56 to 69.13 ng/L and an average concentration of 28.44 ± 16.37 ng/L. Among the detected compounds, PFBA, PFPeA, and PFHxA were the most frequently observed, with mean concentrations of 6.38 ± 4.87 ng/L, 5.45 ± 3.80 ng/L, and 4.98 ± 5.32 ng/L, respectively. Overall, short-chain PFAS exhibited higher concentrations and detection frequencies than long-chain PFAS, and PFCAs showed higher concentrations and detection frequencies than PFSAs (Figure 2a1,b).

The total PFAS concentrations in sediment samples ranged from 1.31 to 17.35 ng/g dw, with a mean concentration of 6.41 ± 4.20 ng/g dw. Among these compounds, PFBA and PFPeA exhibited detection rates of 100%, whereas the detection rates of other PFCA compounds ranged from 78% to 94%. PFSA compounds generally exhibited lower detection rates than PFCAs. In contrast to the distribution characteristics observed in river water, PFAS in sediments were predominantly composed of long-chain compounds (C ≥ 8), with PFSA compounds accounting for a substantially higher proportion than in the surface water (Figure 2a2,b).

The PFAS concentrations in river water and sediments observed in this study were comparable to those reported for natural surface water bodies, such as the Yellow River basin and six major rivers in South Korea [25,26], but were substantially lower than those reported for urban rivers receiving industrial effluents, including those in Las Vegas and the Fuxin Industrial Park [27,28]. Sources of PFAS in urban rivers are complex and include surface runoff, municipal sewage, industrial effluents, and landfill leachate [9,29,30]. Moreover, the magnitude of PFAS contamination in urban rivers is closely associated with urban population density and industrial structure [31]. For instance, along the main stem of the Yangtze River, PFAS concentrations exhibit pronounced spatial variability among urban sections, reflecting differences in industrial intensity and human activity patterns across the cities it traverses [32]. In the Chongqing section of the upper Yangtze River, which is dominated by chemical and machinery manufacturing industries, total concentrations of 16 PFAS ranged from 1.54 to 61.94 ng/L [33]. In the middle reaches at Wuhan, total concentrations of 8 PFAS in the Han River basin ranged from 8.60 to 568 ng/L, likely attributable to local domestic and industrial wastewater discharges [34]. Cities in the lower reaches are characterized by well-developed chemical, textile, and papermaking industries, with PFAS concentrations peaking at 902 ng/L in the Nantong section of the main stem [35]. In this study, urban rivers were primarily derived from the upstream Huangcun and Xiaohongmen WWTPs. Both facilities mainly treat urban domestic sewage, with average PFAS concentrations of 50.0 ng/L in influent and 62.3 ng/L in effluent [36]. In comparison with global data from nearly 500 WWTPs reported by Lenka et al. [13], these values fall within the lower range of the reported concentration distribution.

### 3.2. Spatial Distribution of PFAS

**Figure 3:**
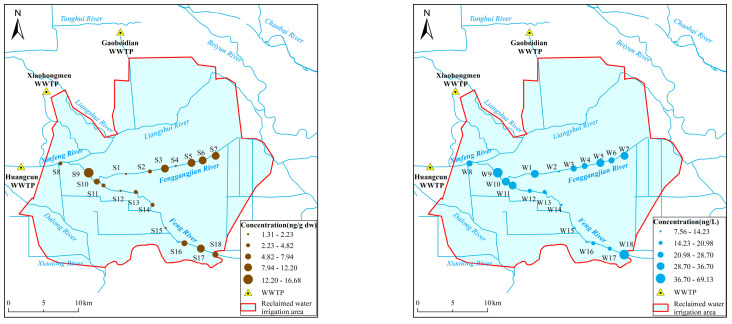
Spatial distribution map of PFAS concentrations in river water (**left**) and sediment (**right**).

In this study, the concentrations of PFAS in the water of the Xinfeng-Feng River were found to be 29.92 ± 19.67 ng/L, while those in the Fenggangjian River were 27.29 ± 6.63 ng/L. The concentrations of sediment PFAS in the Xinfeng-Feng River and Fenggangjian River were found to be 5.89 ± 4.04 ng/g dw and 7.16 ± 4.17 ng/g dw, respectively. The spatial distribution of total PFAS concentrations in river water and sediment is illustrated in Figure 3.

The three primary sources of reclaimed water for the irrigation area are the Gaobeidian, Xiaohongmen, and Huangcun WWTPs. Collectively, these facilities provide almost 300 million m^3^/yr of reclaimed water. The reclaimed water is conveyed to the irrigation area through river channels and irrigation networks for agricultural use. The Xinfeng-Feng River section comprises 10 sampling points. From W8 downstream to W18, a gradual decrease in PFAS concentrations in river water was revealed, indicating the influence of distance from pollution sources [37,38]. The PFAS concentration trends in sediments generally mirrored those observed in the river water. It is noteworthy that the levels of PFAS in the Fenggangjian River are lower than those in the Xinfeng-Feng River, yet the concentrations of PFAS in the sediments are higher than in the Xinfeng-Feng River. This discrepancy is probably because the Fenggangjian River is an artificially excavated channel created by diverting water from the Feng River for irrigation. Its narrow and shallow morphology results in sluggish water flow, which promotes the adsorption of PFAS onto suspended particulate matter and subsequent irregular accumulation in sediments. However, no substantial trend in PFAS concentrations was detected in the Fenggang Jian River. The investigations indicated the presence of a municipal landfill site at monitoring point S5, which may be responsible for the elevated PFAS observed in the vicinity of S5 [39,40].

### 3.3. Sediment–Water Partitioning Characteristics of PFAS

Previous studies indicate that long-chain PFAS exhibit a stronger tendency to adsorb onto sediments, and that, at identical carbon chain lengths, PFSAs show higher sediment adsorption affinity than PFCAs [41,42]. In this study, the sediment–water partition coefficient (*K_d_*) was employed to quantitatively assess this behavior.

*K_d_* = C_s_/C_w_, (1)

where C_s_ is the concentration of PFAS in sediment (ng/g dw), and C_w_ is the concentration of PFAS in water (ng/L).

**Figure 4:**
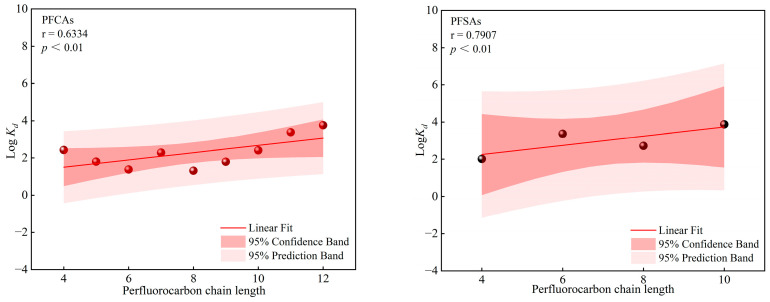
Relationship between Log *K_d_* and chain length of the PFAS.

The sediment–water partition coefficient (Log *K_d_*) of PFAS is strongly influenced by their molecular structure and physicochemical properties, including carbon chain length and functional groups [43,44,45,46]. In this study, the Log *K_d_* of PFAS measured at different locations along the urban rivers increased progressively with carbon chain length, as shown in Figure 4. This trend is consistent with findings reported in previous studies [47]. Owing to the coexistence of hydrophilic and hydrophobic moieties within PFAS molecular structures, increasing carbon chain length results in a higher proportion of hydrophobic groups and enhanced hydrophobicity. Increased hydrophobicity strengthens van der Waals interactions between PFAS molecules and particle surfaces, thereby enhancing their affinity for particulate matter. Meanwhile, aqueous solubility decreases [44], further promoting the preferential adsorption of longer-chain PFAS onto sediments.

### 3.4. Comparison in Different Water Bodies

The average sediment–water partition coefficients (Log *K_d_*) of PFAS in urban rivers receiving reclaimed water, particularly for PFSA compounds, are generally higher than those observed in seawater, natural surface water, and lakes (Table 2).

**Figure 5:**
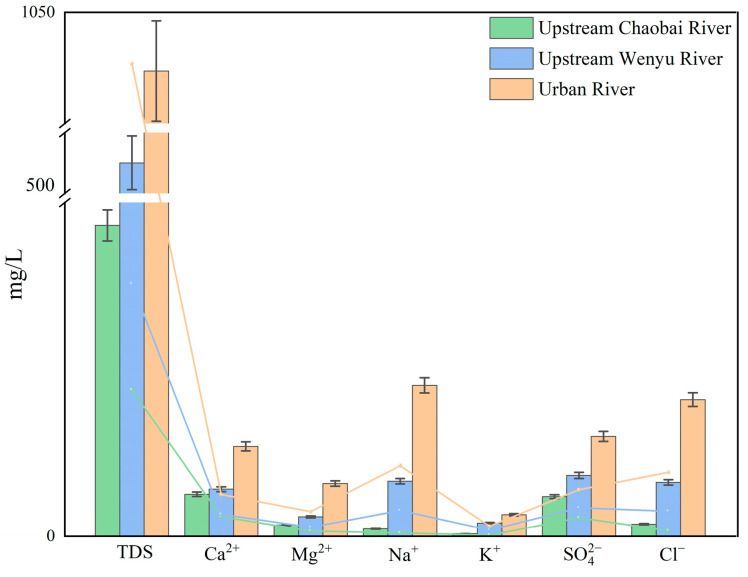
Comparison of chemical parameters of urban and upstream water.

Previous studies indicate that the PFAS distribution in sediment–water systems is influenced not only by carbon chain length and functional groups, but also by the physicochemical properties of the aqueous phase and sediment characteristics [22,48]. When the river contains a high concentration of salt ions such as K^+^, Na^+^, Ca^2+^, Mg^2+^, Cl^−^, and SO_4_^2−^, a potential ‘salting-out effect’ is produced, which encourages the adsorption of PFAS onto sediment surfaces [29,49]. Simultaneously, the effluent contains multiple heavy metal pollutants, including copper (Cu), zinc (Zn), cadmium (Cd), and lead (Pb). Dissolved salt ions and metal cations can reduce the electronegativity of the sediment surface via cation bridging and compression of the electrical double layer, thereby promoting PFAS adsorption onto sediments [29]. As shown in Figure 5, urban water receiving reclaimed water generally exhibits higher total dissolved solids (TDS) and elevated concentrations of salt ions than the upstream surface water [24,50,51]. This fact indicates that urban rivers receiving reclaimed water exhibit distinctive water chemistry characteristics, which may influence the sediment–water partitioning behavior of PFAS. Furthermore, urban rivers, especially within artificially excavated channels, tend to have narrower and shallower channel morphologies than natural waterways. Sluggish or stagnant flow allows suspended particulate matter carrying PFAS to settle and contribute to elevated PFAS Log *K_d_*.

**Table 2:** Statistics on the Log *K_d_* of the dominant PFAS in different types of water.

Water Body Type	Study Area	Sampling Date	PFBA	PFPeA	PFHxA	PFHpA	PFOA	PFNA	PFDA	PFBS	PFHxS	PFOS	Data Sources
Natural surface water	Han River, Korea	2010–2012	/	/	0.02	ND	0.04	0.10	0.13	/	0.03	0.07	[52]
	Qiantang River China	2018.8	1.62	1.71	1.68	1.93	1.96	1.99	2.80	1.46	2.24	2.57	[53]
	Jiulong River, China	2015.8	/	/	1.42	1.73	2.12	1.84	2.40	1.34	1.87	2.25	[54]
Seawater	Korean Coast	2018.5	/	ND	1.70	ND	2.00	ND	ND	ND	1.60	2.50	[25]
	South China Sea	2018.7	0.04	ND	0.15	ND	0.07	1.07	2.22	ND	0.26	0.29	[55]
	Shandong Coast, China	2019.8	1.52	1.25	0.96	2.13	1.67	2.00	2.14	2.15	2.15	2.15	[56]
Lake	Taihu Lake, China	2010.8	1.64	1.29	0.86	1.43	0.65	1.88	2.16	2.24	1.82	2.24	[57]
	Dianchi Lake, China	2010.10	1.18	1.14	1.33	1.33	1.27	1.18	1.64	/	/	1.95	[44]
Urban river	Liao River, China	2012.10	2.03	ND	1.58	2.06	2.14	2.57	ND	2.54	ND	2.31	[58]
	Jiaozhou Bay, China	2018.4	1.09	ND	ND	ND	1.30	1.74	2.22	ND	ND	2.07	[59]
	Xiaoqing River, China	2013.6	0.90	0.70	0.79	0.94	1.19	1.50	2.38	ND	ND	2.02	[60]
	Beijing, China	2021.4	2.42	1.80	1.38	2.28	1.31	1.80	2.40	2.01	3.37	2.60	This study

Note: / indicates not analyzed. ND indicates not detected.

## 4. Conclusions

This study investigated the distribution of PFAS in rivers receiving reclaimed water in suburban Beijing, with a primary focus on the PFAS partitioning characteristics within the sediment–water system and comparisons with other water body types. The results indicate that all 13 target PFAS compounds were detected in both water and sediment samples, with detection frequencies ranging from 6% to 100% in river water and from 11% to 100% in sediments. Short-chain PFAS (C4–C6), predominantly PFCAs, dominated the river water phase, whereas long-chain PFAS and PFSAs accounted for a substantially higher proportion in sediments. From a spatial perspective, PFAS concentrations in the urban runoff exhibited a decreasing trend from the wastewater treatment plant discharge point toward downstream sections as the distance from sewage sources increased.

In this study, the Log *K_d_* of PFAS increased with increasing carbon chain length. The average Log *K_d_* of PFAS in urban rivers are higher than those observed in other surface water systems. This phenomenon may be attributed to the long-term reception of domestic sewage and industrial wastewater by urban rivers. Such inputs lead to distinct water chemistry characteristics, including substantially higher total dissolved solids (TDS), elevated concentrations of inorganic salt ions, and the coexistence of multiple heavy metal ions. Under the combined influence of high ionic strength and mixed metal ions, PFAS are more likely to adsorb onto suspended particulate matter through mechanisms such as charge neutralization, cation bridging, and co-adsorption. Meanwhile, urban runoff channels are generally narrow and shallow with slow or even stagnant flow. This facilitates the settling and accumulation of PFAS-carrying suspended particulate matter in bottom sediments. These processes collectively lead to an increase in the PFAS sediment–water partition coefficient. This paper focuses on the analysis of water chemistry characteristics in different water bodies, while sediment–water partition coefficients are jointly governed by sediment properties and water chemistry. Consequently, sediment properties should also be considered a necessary factor in future research.

## Figures and Tables

**Table 1:** Recoveries and limits of detection (LODs) of PFAS in water and sediment samples.

Compounds	LOD		Mean Recovery (%)	
	Water (ng/L)	Sediment (ng/g dw)	Water	Sediment
PFBA	0.1	0.03	92.0	57.8
PFPeA	0.1	0.02	91.5	60.1
PFHxA	0.2	0.02	89.2	59.5
PFHpA	0.1	0.03	90.6	62.6
PFOA	0.1	0.02	85.9	73.8
PFNA	0.1	0.03	96.1	71.2
PFDA	0.2	0.02	85.3	66.5
PFUnDA	0.2	0.03	82.1	69.7
PFDoDA	0.2	0.03	67.3	62.2
PFBS	0.2	0.02	93.5	65.8
PFHxS	0.1	0.02	95.8	69.5
PFOS	0.2	0.02	96.1	72.0
PFDS	0.2	0.03	75.7	61.7
^13^C_4_PFBA	--	--	70.6	88.0
^13^C_2_PFHxA	--	--	92.4	95.0
^13^C_4_PFOA	--	--	87.2	96.5
^13^C_5_PFNA	--	--	82.1	96.1
^13^C_2_PFDA	--	--	62.7	98.0
^13^C_2_PFUnDA	--	--	59.0	82.3
^18^O_2_PFHxS	--	--	74.4	76.9
^13^C_4_PFOS	--	--	87.0	73.6

## Data Availability

The original contributions presented in this study are included in the article. Further inquiries can be directed to the corresponding author(s).

## Abbreviations

The following abbreviations are used in this manuscript:

PFAS	Per- and Polyfluoroalkyl Substances
WWTPs	Wastewater Treatment Plants
PFCAs	Perfluoroalkyl carboxylic acids
PFSAs	Perfluoroalkyl sulfonic acids
Log *K_d_*	Sediment–water ratio/Sediment–water partition coefficients
